# Kinetic Considerations in the Interpretation of Biomonitoring of 1,3-Butadiene Exposure by Determination of Urinary Mercapturic Acids

**DOI:** 10.3390/toxics12090623

**Published:** 2024-08-23

**Authors:** Peter J. Boogaard, Mary Freire de Carvalho, Maryam Zare Jeddi

**Affiliations:** 1 Division of Toxicology, Wageningen University, P.O. Box 8000, 6700 EA Wageningen, The Netherlands; 2 Shell Health, Shell International B.V., 2596 HR The Hague, The Netherlands; m.freiredecarvalho@shell.com; 3 Shell Global Solutions B.V., 2596 HR The Hague, The Netherlands; maryam.zarejeddi@shell.com

**Keywords:** butadiene, human biomonitoring, toxicokinetics, mercapturic acid, urinary biomarkers, half-life occupational exposure

## Abstract

1,3-Butadiene (BD) is classified as a human carcinogen, and occupational exposure should be minimized. This study examined the effectiveness of personal protective equipment (PPE) during the clean-up and repair of a storage tank containing sludge contaminated with BD. A total of 66 workers participated, providing repeat urine samples before and after the shift. Overall, 1286 samples were analyzed for 1,2-dihydroxy-4-(*N*-acetylcysteinyl)butane (DHBMA) and the isomers 2-hydroxy-1-(*N*-acetylcysteinyl)-3-butene and 1-hydroxy-2-(*N*-acetylcysteinyl)-3-butene (MHBMA). Both DHBMA and MHBMA are urinary metabolites of BD and serve as biomarkers for recent BD exposure. Established correlations between the urinary concentrations of these biomarkers and airborne BD levels allowed for exposure assessment. However, conclusions regarding the exceedances of occupational exposure limits can vary depending on whether DHBMA or MHBMA levels are considered. This study investigated this discrepancy by estimating the apparent urinary half-lives of DHBMA and MHBMA using sequential individual post- and pre-shift samples. The results indicated that the longer urinary half-life of MHBMA (19.7 ± 3.1 h) led to its accumulation during the work week, in contrast to DHBMA, which has a shorter half-life (10.3 ± 1.9 h) and showed limited accumulation. When the kinetic information was used to adjust for the MHBMA build-up over the week, the discrepancy with DHBMA resolved, confirming that exposure limit values were not exceeded and validating the effectiveness of the PPE used. In the context of biomonitoring, this study provides valuable insights into biomarker selection based on specific objectives. MHBMA is recommended for scenarios with uncertain exposure timing and activities, whereas DHBMA is the preferred biomarker for evaluating the effectiveness of protective measures in known exposure settings.

## 1. Introduction

1,3-Butadiene (BD) is an important petrochemical compound produced in high volumes and is primarily used as a major commodity chemical for the manufacture of synthetic rubbers and thermoplastic resins. In addition, BD is present in cigarette smoke, cooking fumes, forest fire smoke, the combustion fumes of plastics, and automobile exhausts [1,2]. BD is one of the top air pollution risk drivers and has long been identified as a rodent carcinogen, showing a very distinct interspecies variation in cancer susceptibility with mice being about two to three orders of magnitude more sensitive than rats. The difference seems to be primarily based on differences in metabolism, and comparison with human metabolism would suggest that humans are less susceptible to BD-induced cancer than rodents [1,2,3]. Nevertheless, BD was classified by the International Agency on the Research on Cancer (IARC) as a category 1 carcinogen, based on sufficient epidemiological evidence suggesting that BD induces lymphohematopoietic cancers in occupationally exposed workers [4]. In the European Union, BD is classified as a category 1A carcinogen and a category 1B mutagen. Consequently, stringent measures are necessary to mitigate occupational exposure to BD. The EU has established binding occupational exposure limits (OELs) for BD to ensure worker safety. The limit for BD is set at 1 ppm (2.2 mg/m^3^) of air, measured as an eight-hour time-weighted average (TWA) (https://echa.europa.eu/substance-information/-/substanceinfo/100.003.138, accessed on 27 May 2024). Additionally, a short-term exposure limit (STEL) of 5 ppm over a 15 min TWA has been adopted. This is particularly relevant in industrial settings where BD is produced, transported, and applied as a building block in the chemical synthesis of polymers.

**Figure 1:**
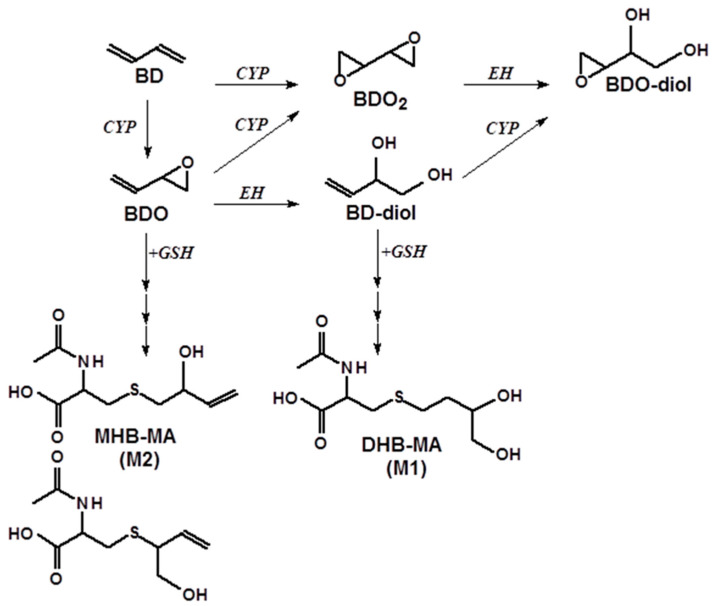
Oxidative metabolism of 1,3-butadiene (BD) and the formation of mercapturic acids.

Occupational exposure to BD is mainly by inhalation, and, once absorbed, it is metabolized through oxidization, hydrolysis, and conjugation with glutathione. The metabolism of inhaled BD and its primary metabolite 1,2-epoxy-3-butene (BDO) has been thoroughly investigated in rodents [5,6]. Apart from a wide range of oxidative metabolites, a number of mercapturic acids were also identified in the urine of exposed animals, including 1,2-dihydroxy-4-(*N*-acetylcysteinyl)butane or dihydroxybutane mercapturic acid (DHBMA or M1) and two isomers of the mercapturic acid of monohydroxybutene: 2-hydroxy-1-(*N*-acetylcysteinyl)-3-butene and 1-hydroxy-2-(*N*-acetylcysteinyl)-3-butene (MHBMA or M2) [5,6]. Both DHBMA and MHBMA result from the conjugation of the epoxides of BD with glutathione and subsequent further metabolic processing and are readily excreted into the urine (Figure 1). Both mercapturic acids were identified as suitable biomarkers for exposure assessment of BD, with DHBMA accounting for more than 97% of the total urinary BD metabolites in humans [7,8,9]. DHBMA (high sensitivity) and MHBMA (high specificity) have been successfully used as urinary biomarkers of exposure to BD in transitional epidemiological studies within the rubber manufacturing industry [7,10].

In our operations, exposure to BD is routinely assessed through human biomonitoring, using hemoglobin adducts in blood and measuring DHBMA and MHBMA in urine [11]. The choice of biomarker is linked to the exposure scenario being investigated. Hemoglobin adducts are primarily relevant for monitoring over longer periods—up to three months—during which potential exposure events may not be exactly known. In contrast, urinary markers are more indicative of recent exposure, such as what might occur during a particular work shift [8,12,13]. The previously determined correlation between airborne exposures to BD and the urinary excretion of mercapturates is used to interpret the results [13]. This correlation between external and internal exposures is widely used as it formed the basis for the EKA (exposure equivalents for carcinogenic substances) values as published by the German MAK Committee [14]. However, we have found that in practical applications, different conclusions could be drawn from the same urine sample concerning the assumed exceedance of the limit values when comparing the DHBMA and MHBMA concentrations in this urine sample. Therefore, the aim of the current study is to investigate potential causes for these consistently observed discrepancies.

## 2. Methods

### 2.1. Study Population and Sample Collection

This study was carried out as part of a biomonitoring campaign at a chemical plant involved in the production, storage, and handling of BD. The study included 66 participants engaged in a clean-up and repair operation of storage tanks. Although the tanks had been taken out of service, they might still contain residues with BD present, particularly in sludge. Given the circumstances, exposure control relies heavily on the use of personal protective equipment (PPE), including the use of chemical-impervious suits and independently supplied breathing air. The workers participating in the study wore these PPE during their work shifts to mitigate exposure risks. Potential exposure to BD could still occur due to the improper fit of the PPE, the migration of BD through the PPE, or external contamination during donning and doffing, particularly during breaks and at the end of shifts.

To evaluate the effectiveness of PPE for short-term exposure, urine-based biomonitoring was undertaken. Urinary samples were collected on a voluntary basis, with each of the 66 participating workers being asked to provide samples at both the beginning and the end of their shifts. The study design allowed for the collection of paired pre- and post-shift samples from individual workers over consecutive days, with some workers providing samples for up to five days. This resulted in a total of 1286 samples, including multiple paired samples from each worker. Instances of missing pre- or post-shift samples were addressed through data imputation.

### 2.2. Measurement of Urinary MHBMA and DHBMA

Urine samples were prepared and analyzed for DHBMA and MHBMA following a previously described protocol, albeit with modification: methylation was omitted and UPLC was utilized instead of GC [8,13]. Briefly, 1.0 mL aliquots of urine were acidified with 50 µL of concentrated formic acid to achieve a pH of approximately 2.5 and then were homogenized. To these samples, 10 µL of a 100 mg/L solution containing both [d_7_]-DHBMA and [d_6_]-MHBMA as internal standards was added, followed by vigorously mixing for approximately 15 s. Samples were then applied to a preconditioned Strata^®^-X (Phenomenex, Aschaffenburg, Germany) solid phase extraction column and passed through under a slight vacuum. The columns were subsequently washed with 2 mL of 0.1% formic acid and subsequently dried under slight vacuum. The analytes were eluted with 2 mL of an ethyl acetate/acetone mixture (2:1 *v*/*v*), collected into a test tube, and concentrated to approximately 0.5 mL by vacuum centrifugation. Finally, the residues were dissolved in 1 mL of 0.1% formic acid and transferred into a 2 mL autosampler vial for analysis.

The analyses were conducted using a Waters UPLC-MS-MS system (Waters, Eschborn, Germany), which included a Waters Acuity UPLC and a Waters TQD MS-MS detector. For the analysis, a 10 µL volume of the prepared sample was injected onto a Waters Acuity UPLC HSS C18 column (2.1 × 100 mm, 1.8 µm). The sample was eluted with a linear gradient of Eluent A consisting of 0.1% aqueous formic acid and Eluent B, consisting of 0.1% formic acid in methanol. The oven temperature was set at 50 °C. The specific gradient and flow rate parameters used for the elution are detailed in Table 1.

Mass fragmentation and detection were performed using negative electrospray-ionization (ESI) mode for DHBMA and positive ESI mode for MHBMA. The retention times and fragments for both mercapturates are listed in Table 2. Instrument processing and data handling were managed using the instrument’s software, Mass Lynx 4.1 (Waters, Eschborn, Germany). Calibration curves were generated in control urine samples spiked with concentrations ranging from 0–20 mg/L for DHBMA and 0–200 µg/L for MHBMA. The results were adjusted for variations in urinary volume by assessing creatinine levels, which were measured according to the Jaffe method [15]. The limits of quantification (LOQs) for DHBMA and MHBMA, were 1 µg/L and 0.1 µg/L urine, respectively.

### 2.3. Data Preparation and Missing Data Imputation

A total of 1286 urinary samples were collected and analyzed from the 66 participating workers, each providing samples both before and after their shifts. However, there were instances where either a pre- or post-shift sample was missing. In some cases, two pre- or post-shift samples were collected on the same day, and the average of these measurements was used. There were 384 instances where a measurement pair lacked either a pre- or post-shift sample. For these missing values, the average of the pre- or post-shift measurements from the preceding and following days was calculated. Additionally, 27 workers provided only a single pre- or post-shift sample without further samples from other days; these cases were excluded from the analysis. After imputing the missing pre- or post-shift measurements, the final analysis included 820 complete measurement pairs, contributing to 1640 datapoints in total. The imputation process resulted in a minimal change to the log distribution of differences between the pre- and post-shift measurements, which was statistically not significant.

### 2.4. Statistical Analysis

In this study, Student’s *t*-test was utilized to statistically compare the mean concentrations of BD metabolites (DHBMA and MHBMA) in urine samples collected from workers before and after their shifts. This comparison aimed to determine if exposure during the shift led to a statistically significant increase in metabolite levels, thereby assessing the effectiveness of the PPE. Therefore, Student’s *t*-test at the 1% significance level was applied to assess the differences for both urinary DHBMA concentrations between samples collected pre- and post-shift and for urinary MHBMA concentrations between samples collected pre- and post-shift.

### 2.5. Estimation of 1,3-Butadiene Air Concentration

The airborne concentration of BD was calculated using the equations previously derived for the two metabolites [13]:

For MHBMA: log10(MHBMA) = log10(BD_air_) + 1.6021

For DHBMA: log10(DHBMA) = 0.882 ∗ log10(BD_air_) + 3.2246

where MHBMA and DHBMA represent the concentrations of the metabolites expressed in µg/g creatinine, and BD_air_ represents the BD concentration in the air expressed in ppm. After processing the data, the airborne concentration during the shift hours for each study participant was calculated using these equations. It is important to note that for MHBMA, urinary metabolite levels below 1.5 µg/g creatinine were excluded from the calculations as they fall below the quantification limit for this metabolite.

### 2.6. Half-Life Estimation

In this study, first-order kinetics were assumed for the metabolism of BD. Kinetic studies conducted in mice and rats have indicated that BD metabolism follows first-order kinetics at exposure concentrations below 1000 ppm, a range well within the expected exposures in this study [5,16,17].

To calculate the half-life of the BD metabolites, we utilized the following exponential decay equation:

$$T_{1/2}=\frac{Time}{ln(0.5)(\frac{C\left(1\right)}{C(0)})}$$

*T*_1/2_ = Half-life (h)

Time = Time elapsed from end of shift to beginning of next work shift (h)

*C*(1) = End-of-shift concentration (µg/g creatinine)

*C*(0) = Pre-next-shift concentration (µg/g creatinine)

The half-life was calculated using the concentration at the end of the last shift (*C*(1)) and the concentration at the beginning of the next work shift (*C*(0)), with time set as the total hours between these two points. Half-life calculations that resulted in a lower final concentration (*C*(1)) than the initial concentration (*C*(0)) were excluded from the analysis. Additionally, for inclusion in the half-life calculations, there was a requirement that the percent change from the initial (*C*(0)) to final (*C*(1)) concentration exceed 15%. This cutoff allows the inclusion of datapoints within 2 standard deviations of the mean, effectively excluding extreme values that were more than 2 standard deviations from the mean. These procedures helped to normalize the data by eliminating biologically non-relevant half-lives, which accounted for approximately 10% of the total dataset, yet still maintaining a sufficiently large dataset for robust analysis.

### 2.7. MHBMA Urinary Correction

The data collected on the urinary concentrations of both mercapturates showed a substantially longer apparent urinary half-life for MHBMA than for DHBMA, suggesting a potential accumulation of MHBMA over time. To correct for this buildup of the background level between shifts and to enable a more accurate prediction of airborne BD exposure, a modification factor for the measured MHBMA data was calculated. We designated the first urinary sample collected from each participant as the background concentration for that individual. This value was then subtracted from all subsequent measurements for the same individual to represent the MHBMA background concentration, effectively factoring out the influence of sampling without breaks to allow for a return to baseline levels. This adjustment allowed us to account for individual variations in MHBMA buildup. The adjusted dataset was subsequently used to estimate the corresponding airborne BD exposure levels.

## 3. Results

### 3.1. MHBMA in Pre- and Post-Shift Urine Samples of Butadiene Factory Workers

Table 3 provides an overview of the mean and range values of MHBMA in pre- and post-shift urine samples. The values for pre-shift MHBMA measurements ranged from 5.6 to 248.9 µg/g creatinine, with a mean of 32.5. Post-shift samples for MHBMA showed a range from 6.0 to 205 µg/g creatinine, with a mean of 37.8.

**Figure 2:**
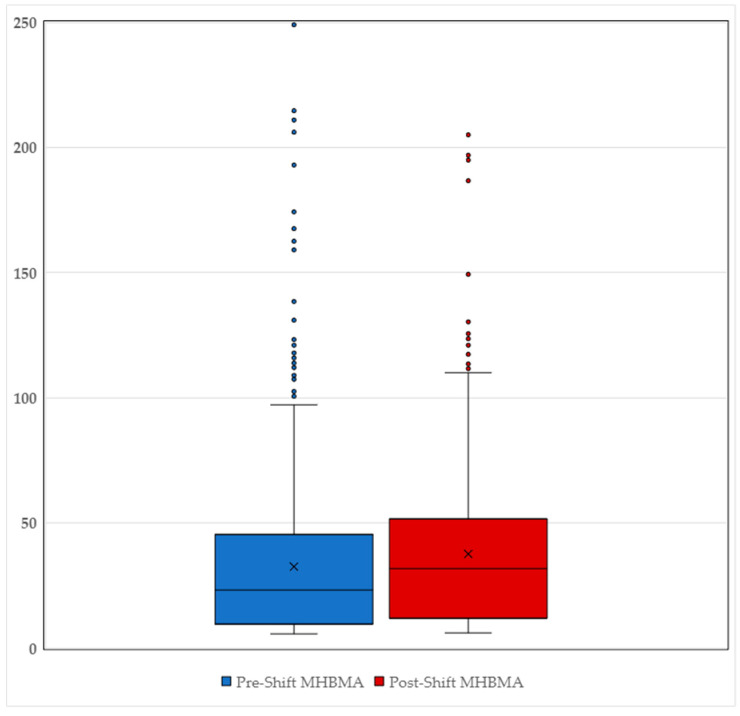
Distribution of pre- (blue) and post-shift (red) MHBMA urine samples.

The increase in MHBMA concentrations from pre- to post-shift was statistically significant, as determined by Student’s *t*-test at a 1% significance level. The box and whisker plot in Figure 2 illustrates the distribution of MHBMA urine concentration measurements in both pre- and post-shift samples, highlighting the wide distribution of values at the upper end of the dataset. Notably, 95% of all datapoints were contained within the range of 5.6–92.6 µg/g creatinine for pre-shift samples and 6.1–112.0 µg/g creatinine for post-shift samples.

### 3.2. DHBMA in Pre- and Post-Shift Urine Samples of BD Factory Workers

**Figure 3:**
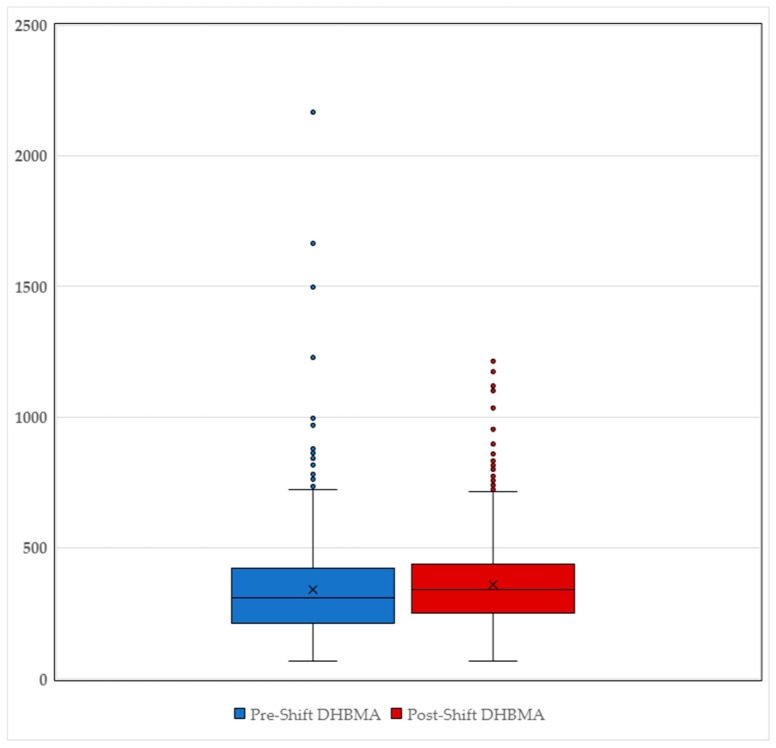
Distribution of pre- (blue) and post-shift (red) DHBMA urine samples.

Table 3 provides an overview of the mean and range values of DHBMA in pre- and post-shift urine samples. The concentrations of DHBMA ranged from 66 to 2166 µg/g creatinine, illustrating a wide variability in the data with some extreme values at the upper end of the distribution. The mean concentrations of DHBMA were 342.1 µg/g creatinine for pre-shift samples and 361.6 µg/g creatinine for post-shift samples. This difference was statistically significant, as determined by Student’s *t*-test, with significance at the 1% level. The box and whisker plot in Figure 3 shows the distribution of the pre- and post-shift DHBMA concentrations. Notably, 95% of the datapoints fell within the range of 74 to 722 µg/g creatinine, suggesting substantial exposure variations among workers and a significant increase in DHBMA levels following shifts.

### 3.3. Half-Life Estimation

**Figure 4:**
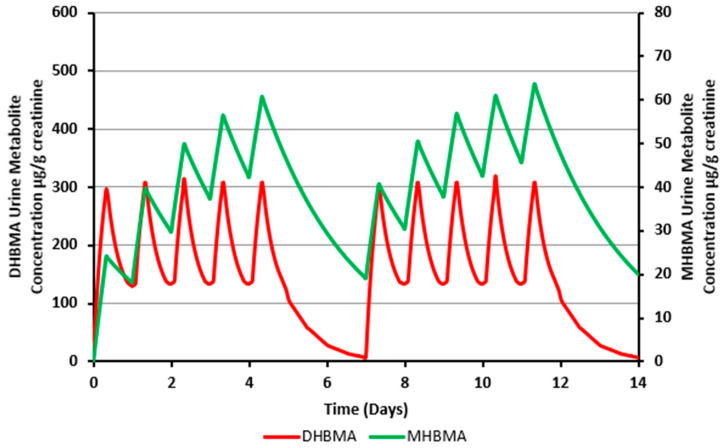
Urinary MHBMA and DHBMA concentrations in a hypothetical worker exposed to an identical concentration of BD each day, during a 5-day work shift over a period of two weeks. The green line represents urinary MHBMA concentration predictions, and the red line represents urinary DHBMA concentration predictions.

Based on the urinary metabolite concentrations, an average apparent urinary half-life of 19.7 ± 3.1 h was calculated for MHBMA and an average apparent urinary half-life of 10.3 ± 1.9 h for DHBMA. Figure 4 shows the BD exposure predictions for a hypothetical worker over the course of two weeks, based on both MHBMA and DHBMA concentrations. The graph demonstrates a gradual increase in the predicted urinary MHBMA concentration throughout the work week, indicative of the accumulation of the individual’s background level. It also shows a decline during the 2-day break during the week; however, the levels do not completely return to baseline, highlighting the cumulative effect of continuous exposure in the case of MHBMA. In contrast, urinary DHBMA levels remain consistent throughout the work week and completely return to background levels during the 2-day break in the week. This contrast underscores the differences in metabolic behavior and clearance rates between these two metabolites.

### 3.4. Estimation of 1,3-Butadiene Air Concentration

Based on previous studies performed by van Sittert et al. [13] which established a relationship between airborne BD concentrations and urinary mercapturic acids in spot urine samples, this study applied the reported linear regression to the current dataset of urinary samples to calculate the corresponding airborne BD levels. The linear regression equations used for these estimations are detailed in the Section 2.

**Figure 5:**
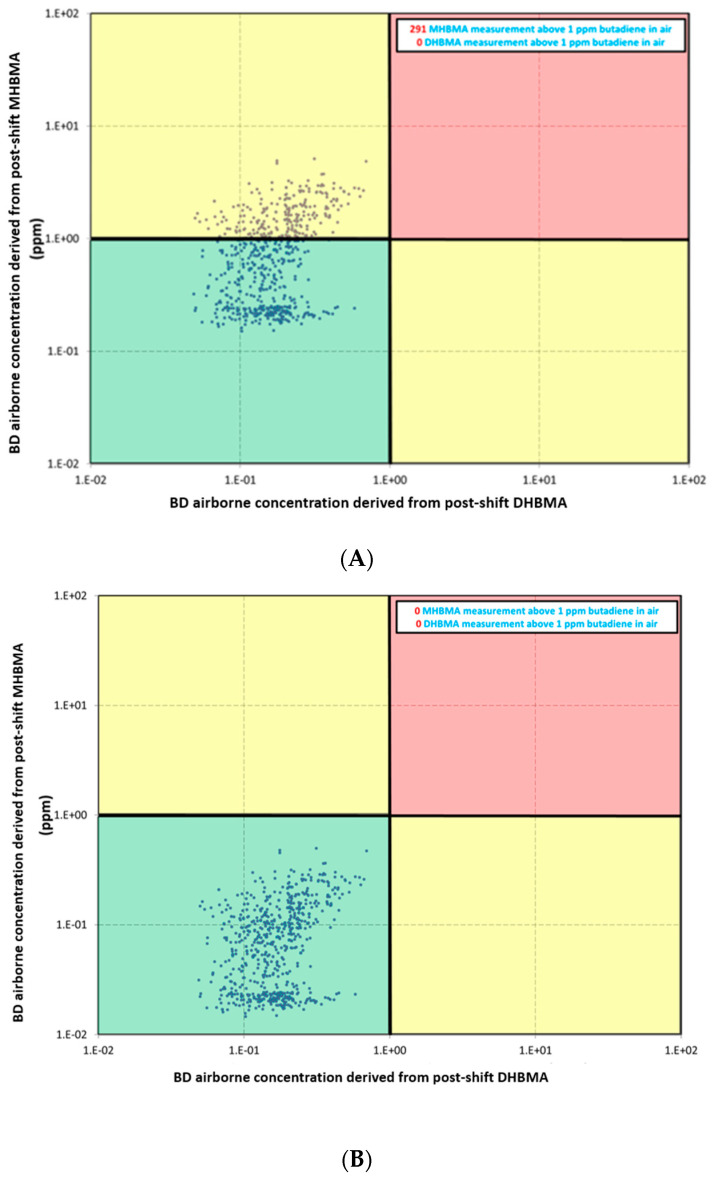
(**A**): Graphical representation of derived airborne concentrations of BD calculated from post-shift urine concentrations of MHBMA and DHBMA. (**B**): Graphical representation of derived airborne concentrations of BD calculated from post-shift urine concentrations of MHBMA and DHBMA after correction for build-up of MHBMA using its apparent urinary half-life. The bold lines represent the value of 1 ppm. Exceedances of 1 ppm derived from urinary MHBMA or DHBMA concentrations are found in yellow quadrants, levels under 1 ppm are found in the green quadrant, and exceedances of 1 ppm from both urinary DHBMA and MHBMA concentrations are found in the red quadrant.

Figure 5A,B display the estimated airborne BD concentrations at the time of exposure, based on the paired datapoints from individual pre- and post-shift urine concentration measurements of MHBMA and DHBMA. The bold lines in these figures represent an airborne exposure of 1 ppm BD. The vertical axis indicates airborne exceedances of 1 ppm based on MHBMA, while the horizontal axis shows exceedances of 1 ppm based on DHBMA. Datapoints in the green quadrant represent no predicted exceedances of 1 ppm threshold. The yellow quadrants indicate a 1 ppm exceedance for either MHBMA or DHBMA, while the red quadrant shows a 1 ppm exceedance derived from both urinary DHBMA and MHBMA concentrations.

Figure 5A shows the estimated BD airborne concentration based on post-shift metabolite measurements. The majority of the datapoints are below the 1 ppm BD threshold. There were 291 measurements based on MHBMA concentrations that exceed 1 ppm BD, but no 1 ppm exceedances were observed based on DHBMA concentrations.

Figure 5B shows the estimated BD airborne concentrations based on post-shift metabolite measurements after correcting the MHBMA data for background concentration build-up due to its relatively long apparent urinary half-life, which can lead to higher than expected BD airborne predictions. When the initial background concentration measurement for urinary MHBMA was corrected for each individual, as described in the Methods, the resulting dataset no longer showed any 1 ppm exceedances based on MHBMA, thus confirming the effectiveness of the adopted exposure control measures. This adjustment significantly enhanced the accuracy of BD exposure assessment, highlighting the importance of considering metabolic half-life in the analysis of biomonitoring data.

## 4. Discussion

Given the potential carcinogenicity of BD in humans, it is important to minimize exposure to BD to the greatest extent possible. In occupational settings where BD is produced or handled, efforts should be made to reduce exposure and to routinely monitor it to ensure compliance with established limit values. Using a previously established method [8,13], urinary mercapturates (MHBMA and DHBMA) were determined in a worker population with potential exposure to BD. The measured values were used to estimate the corresponding airborne exposure to BD. Initial analysis indicated numerous exceedances of the occupational exposure limit of 1 ppm using urinary MHBMA concentrations, in contrast to essentially no 1 ppm exceedances when urinary DHBMA concentrations from the same urine sample were considered. The objective of this investigation was to identify the cause for this apparent discrepancy.

The normal benchmark values for background concentrations of MHBMA and DHBMA are <2 and 400 µg/g creatinine, respectively [18]. The biological limit values (BLVs), which correlate with an occupational exposure to 1 ppm BD as an 8 h TWA, are set at 40 µg/g creatinine for MHBMA and 1600 µg/g creatinine for DHBMA [18]. It is important to note that tobacco smoke contains BD, and its presence is detected through the measurement of both mercapturates [18]. In a recent study, median background values (with the 95% confidence interval) for DHBMA have been reported as 365 µg/g creatinine (347–388) for smokers (n = 884) and 265 (252–277) µg/g creatinine for non-smokers (n = 1283) [19]. These median values were very close to the geometric means, indicative of an apparently normal distribution of the values. For MHBMA, no background was found in either smokers or non-smokers for the isomer 1-hydroxy-2-(*N*-acetylcysteinyl)-3-butene, but for the other isomer, 2-hydroxy-1-(*N*-acetylcysteinyl)-3-butene, a median background (95% confidence interval) of 1.46 (1.18–1.70) µg/g creatinine was measured in smokers (n = 884), while in non-smokers, the concentrations were below the limit of detection of 0.7 µg/L [19]. The geometric means and most of the values in the current dataset fall within the range of benchmark values and BLVs of each metabolite, except for some datapoints at the upper end of the dataset that account for less than 10% of all datapoints.

The MHBMA concentrations measured in both pre- and post-shift samples showed several datapoints (1.7%) at the upper end of the data distribution that are significantly higher than the bulk of the datapoints. While the reason for this observation is unknown, potential contributing factors such as smoking status [20], ethnic background, and genetic polymorphisms [21,22] have been identified in the literature as influencing higher urinary MHBMA levels. Although these factors might partially explain to some extent the elevated background concentrations of MHBMA, they do not fully account for most of the high values observed in the pre-shift urine samples. One possible factor contributing to increased urinary MHBMA concentrations could be its accumulation over time due to a relatively long half-life in comparison to the duration of the shift. Previous studies on S-phenylmercapturic acid (S-PMA), a biomarker for benzene exposure, have shown that apparent urinary half-lives for mercapturic acids can be accurately estimated from large datasets without a specific study design [23,24]. Given that the current dataset included 1286 samples from 65 workers, it was deemed sufficiently robust for this analysis. The calculated apparent urinary half-lives were 19.7 ± 3.1 h for MHBMA and 10.3 ± 1.9 h for DHBMA. This considerable difference in half-lives could explain the discrepancy in the estimated airborne BD concentrations, where values above 1 ppm were initially predicted based on urinary MHBMA concentrations, while the DHBMA measurements in the same urine sample did not indicate such exceedances. As depicted in Figure 4, a significant build-up of MHBMA is observed due to its longer estimated half-life. In contrast, any build-up of DHBMA is largely cleared during non-working periods, with virtually all DHBMA being eliminated over the weekend. This results in an overestimation of BD exposure based on MHBMA concentrations during the work week. However, when the kinetic information was applied to adjust for background concentrations of MHBMA due to build-up, a more accurate MHBMA-based prediction of BD airborne levels was obtained. As shown in Figure 5, the predictions based on the corrected MHBMA values aligned with those based on the urinary DHBMA measurements from the same samples.

The current study underscores the importance of careful data evaluation, considering all the toxicokinetic properties of different metabolites of an investigated chemical. This current study highlights how an initial understanding of the accumulation of MHBMA over time is crucial; without this consideration, BD exposure could be significantly overestimated when directly based on unadjusted urinary measurements. For example, the uncorrected data in Figure 5A suggest that almost half of the workers were exposed to levels exceeding 1 ppm of BD. However, once the background accumulation is accounted for, these apparent 1 ppm exceedances do not occur anymore.

Furthermore, this study raises the question about the validity of the equations used to estimate airborne BD concentrations based on the urinary concentrations of MHBMA and DHBMA [14]. These equations were developed in earlier studies involving a different worker population engaged in the production of styrene-butadiene rubber [8,13]. In those studies, spot urine samples were collected before and after the shifts on days with expected exposure to BD. Since these days were rarely consecutive, it is probable that little to no accumulation of BD metabolites occurred, contrasting with the conditions observed in the current study setting. These differences emphasize the importance of considering the toxicokinetics of biomarkers in relation to the occupational setting and possible exposure scenarios when deriving exposure-biomarker equations.

It is important to acknowledge that while the correction applied to the MHBMA datapoints for the background build-up allows for a more precise prediction of airborne BD exposure, a bulk correction using a single factor might obscure individual variations in toxicokinetic dynamics, such as genotypes that affect the metabolism of MHBMA and DHBMA. These individual differences could significantly influence the metabolism and clearance rates of these metabolites.

A potential limitation of this study is the absence of direct ambient BD concentration measurements at the worksite. Since PPE was worn throughout the study, it relied on urinary biomarker data (MHBMA and DHBMA) to estimate airborne BD exposure levels. While this approach is supported by established correlations between urinary biomarkers and airborne BD concentrations, these correlations may not fully capture site-specific exposure dynamics. Ambient measurements would provide complementary information on the workers’ external exposure, accounting for spatial and temporal variations. Despite these limitations, this study provides valuable insights into occupational BD exposure and the effectiveness of PPE. Future studies should consider including direct ambient BD measurements to enhance the accuracy and validation of biomarker-based exposure assessments.

In the context of biomonitoring activities, our study provides valuable insights into biomarker selection for specific objectives. For general surveillance of BD exposure, urinary MHBMA is the preferred biomarker due to its longer half-life, which can detect BD exposure over extended periods without necessarily accounting for fluctuating background levels that may remain in urine. For instance, urinary MHBMA would be the preferred biomarker in settings where exposure to BD is anticipated or suspected but the specific conditions or peak exposure periods are not well defined.

Conversely, DHBMA, with its shorter half-life, is more suited for associating certain activities or shift work to BD exposures. Collecting urinary DHBMA at the end of the shift, as previously suggested [8,13,14], offers a more immediate reflection of BD exposure linked to specific operations or tasks performed during that shift. This makes DHBMA an ideal biomarker for verifying the effectiveness of protective measures in place during known exposure scenarios. Therefore, the choice of biomarker—MHBMA or DHBMA—for BD exposure assessment should be guided by the specific exposure assessment goals and the occupational setting.

This study has several important implications for occupational health and safety, particularly concerning the monitoring and management of exposure to 1,3-butadiene (BD) in industrial settings. This study demonstrates the effectiveness of using urinary biomarkers (MHBMA and DHBMA) for assessing occupational exposure to BD. It highlights the different kinetic behaviors of these biomarkers, with MHBMA showing a longer half-life and accumulation over time compared to DHBMA. This information is crucial for selecting appropriate biomarkers for specific monitoring scenarios.

The biomonitoring of BD can be effectively used for the validation of personal protective equipment (PPE). Additionally, this study provides valuable insights into designing biomonitoring strategies. It suggests that MHBMA is more suitable for monitoring exposure over extended periods or uncertain exposure timings, while DHBMA is better for immediate post-exposure assessments. This approach ensures that biomonitoring not only serves as a tool for exposure assessment but also enhances the implementation of appropriate control measures to mitigate the health risks associated with BD exposure. This guidance can help occupational health professionals tailor monitoring programs to specific workplace conditions and exposure scenarios.

Overall, these findings suggest areas for future research, including the need for studies that combine biomarker monitoring with direct ambient measurements and investigate the impact of individual variability on biomarker levels. Such research can further refine exposure assessment methods and enhance worker protection strategies.

## Figures and Tables

**Table 1:** Gradient and flow.

Time	Eluent A (%)	Eluent B (%)	Curve	Flow (mL/min)
0	95	5	6	0.35
5	5	95	6	0.35
5.1	95	5	6	0.35
7	95	5	6	0.35

**Table 2:** Retention times and mass fragments.

Analyte	Retention Time (min)	Parent Fragments	Daughter Fragments	ESI-Mode
DHBMA	1.53	250.06	120.83	negative
[d_7_]-DHBMA	1.51	257.06	127.94	negative
MHBMA	2.12	234.07	161.92	positive
[d_6_]-MHBMA	2.05	240.07	161.99	positive

**Table 3:** Urinary concentrations of MHBMA and DHBMA in BD factory workers * (N = 820).

Work Shift	MHBMA	MHBMA	DHBMA	DHBMA
	Mean	Range	Mean	Range
Pre-shift	32.5	5.6–248.9	342.1	66–2166
Post-shift	37.8	6.0–205	361.6	69–1214

* All values reported in µg/g creatinine.

## Data Availability

The data supporting the conclusions of this article will be made available by the authors on request.
